# Characterization and Crystal Structure of a Robust Cyclohexanone Monooxygenase

**DOI:** 10.1002/anie.201608951

**Published:** 2016-11-22

**Authors:** Elvira Romero, J. Rubén Gómez Castellanos, Andrea Mattevi, Marco W. Fraaije

**Affiliations:** ^1^ Department of BiotechnologyUniversity of GroningenNijenborgh 49747AGGroningenThe Netherlands; ^2^ Department of Biology and Biotechnology “Lazzaro Spallanzani”University of PaviaVia Ferrata 927100PaviaItaly

**Keywords:** Baeyer–Villiger oxidation, biocatalysis, cyclohexanone monooxygenase, ϵ-caprolactone, enzyme stability

## Abstract

Cyclohexanone monooxygenase (CHMO) is a promising biocatalyst for industrial reactions owing to its broad substrate spectrum and excellent regio‐, chemo‐, and enantioselectivity. However, the low stability of many Baeyer–Villiger monooxygenases is an obstacle for their exploitation in industry. Characterization and crystal structure determination of a robust CHMO from *Thermocrispum municipale* is reported. The enzyme efficiently converts a variety of aliphatic, aromatic, and cyclic ketones, as well as prochiral sulfides. A compact substrate‐binding cavity explains its preference for small rather than bulky substrates. Small‐scale conversions with either purified enzyme or whole cells demonstrated the remarkable properties of this newly discovered CHMO. The exceptional solvent tolerance and thermostability make the enzyme very attractive for biotechnology.

Cyclohexanone monooxygenase (CHMO; EC 1.14.13.22) is an FAD‐ and NADPH‐dependent Baeyer–Villiger monooxygenase (BVMO).^1^ A wide variety of ketones are converted by CHMO into esters or lactones through the insertion of an oxygen atom on one side or the other of the carbonyl group. In addition, CHMO oxidizes aldehydes and heteroatoms^2^ and carries out epoxidation reactions.^3^ In contrast to conventional Baeyer–Villiger oxidations using peracids, CHMO reactions are environmentally friendly and often proceed with excellent regio‐, chemo‐, and enantioselectivity.^4^ Numerous industrial applications have been suggested for CHMO.^5^ The conversion of cyclohexanone as catalyzed by CHMO is of particular interest since the product, ϵ‐caprolactone, is a precursor of both adipic acid and ϵ‐caprolactam,^6^ which are known polymer building blocks.^7^ One of the main barriers to exploiting CHMO as a biocatalyst is its lack of stability.^8^ To date, the best‐characterized CHMOs are from *Acinetobacter calcoaceticus* NCIMB 9871 (AcCHMO) and *Rhodococcus* sp. HI‐31 (RhCHMO). Similar CHMOs have been identified from various bacteria (Table S1 in the Supporting Information). However, engineering attempts to improve their stability have met with limited success.^9^ The only robust BVMO described so far, phenylacetone monooxygenase from *Thermobifida fusca* (TfPAMO; EC 1.14.13.92), shows no activity on cyclohexanone.^10^ Our interest in using a robust BVMO to produce ϵ‐caprolactone and other valuable compounds prompted us to search for a stable CHMO in the available genomes of thermophiles. Among the retrieved sequences, a putative CHMO from *Thermocrispum municipale* DSM 44069 was found (TmCHMO; NCBI RefSeq: WP_028849141.1). This thermophilic organism was isolated from municipal waste compost.^11^ TmCHMO clusters with known CHMOs sequences (Figures S1, S2 in the Supporting Information), the closest homologue being from *Brachymonas petroleovorans* (66 % sequence identity). We identified three genes upstream of the TmCHMO gene that may participate in cyclohexanol degradation (Table S2). These sequence analyses suggested that the *T. municipale* enzyme may be a robust CHMO.

Therefore, we purified TmCHMO and studied its substrate acceptance (Figure S3).^4^, ^12^ For the sake of comparison, the same analysis was performed with AcCHMO. Small aliphatic ketones such as cyclohexanone, cyclobutanone, and bicyclo[3.2.0]hept‐2‐en‐6‐one were efficiently converted by both CHMOs, although in all cases, the *K*_m_ values for AcCHMO were higher than those measured for TmCHMO, despite similar *k*_cat_ values (Table S3). Conversely, the bulkier molecules progesterone and cyclopentadecanone were not substrates of either enzyme.^13^ At high substrate concentrations, substrate inhibition was observed for TmCHMO, which is not unprecedented for this enzyme class (Figure S4). To avoid decreased conversions as a result of this effect, reaction media with two phases have been successfully implemented.^5^ The uncoupling rate for both CHMOs was around 0.1 s^−1^. To compare their thermostability, the ThermoFAD method was used as a first approach.^14^ This confirmed that AcCHMO is only moderately stable since it exhibits a *T*_m_ value of 37 °C (Table S4).9a,9b Conversely, the *T*_m_ value for TmCHMO was found to be 11 °C higher. Next, the two CHMOs were probed for their resistance to thermal inactivation (Figure 1 A). This revealed that TmCHMO still displayed 58 % residual activity after 5.5 h at 45 °C. By contrast, AcCHMO lost its activity within a few minutes at 45 °C. These experiments were complemented by analyzing the effect of cosolvents on the stability, since enzymes working in organic solvents or aqueous/organic mixtures are often desired for biotechnology.^15^ While AcCHMO essentially lost its activity after 25 min incubation in 14 % acetonitrile at 20 °C, TmCHMO remained highly active (>80 %) for at least 20 h under these conditions (Figure 1 B). These thermostability and solvent‐tolerance data clearly show that TmCHMO is a substantially more robust biocatalyst than AcCHMO.

**Figure 1:**
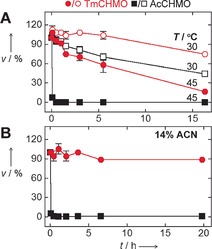
Effect of temperature and acetonitrile (ACN) on the stability of TmCHMO and AcCHMO. Activities on cyclohexanone were measured.

Besides influencing stability, the reaction medium can also modify enzyme selectivity.^15^, ^16^ The effect of cosolvents (Table S5) was determined by using 2‐butanone, the conversion of which to two possible regioisomers is of industrial interest (Scheme S1).^17^ All reactions were stopped after 48 h at 17 °C. The ratio between the products methyl propanoate and ethyl acetate was found to be about 3:7 for both purified TmCHMO and AcCHMO in the absence of any cosolvent (Figure S5). The same regioisomer ratio was observed using whole cells of *Escherichia coli* expressing one or the other CHMO (not shown). Next, we inspected the effect of various solvents at 15 % concentration. The two purified CHMOs exhibited similar results, although the yields of TmCHMO were generally higher. The strongest effect on regioselectivity was observed with 2‐methyl‐1,3‐dioxolane, which led to almost exclusive production of ethyl acetate. 1,3‐Dioxane and 1,4‐dioxane had a more moderate influence, since about 40 % of the total product was methyl propanoate. We also carried out reactions with 30 % methanol or ethanol. These cosolvents had a negligible effect on enzyme regioselectivity. However, they considerably decreased the 2‐butanone conversion yield for AcCHMO (<4 %), while that for TmCHMO remained high (96 % and 56 % for methanol and ethanol, respectively). From these results, it can be concluded that the robustness of TmCHMO makes possible to modulate its regioselectivity through using cosolvents.

Along the lines of the previous experiments, the potential of TmCHMO as a regioselective biocatalyst was further probed by using *rac*‐bicyclo[3.2.0]hept‐2‐en‐6‐one. This compound can be converted by BVMOs into four products (Scheme S2). This reaction is widely used to study the ability of BVMOs to carry out the kinetic resolution of racemic compounds,^18^ and it is of interest for the synthesis of prostaglandins, for example.^19^ Small‐scale conversions of *rac*‐bicyclo[3.2.0]hept‐2‐en‐6‐one were carried out with TmCHMO, AcCHMO, and TfPAMO. Both enantiomers of this ketone were fully converted by both CHMOs, yielding almost exclusively one regioisomer from each enantiomer (Figure S6A and Table S6).^20^ By contrast, TfPAMO produced all four possible lactones, proving to be far less regioselective than CHMOs. We also used the same BVMOs to produce enantiomerically pure sulfoxides, which are widely used in asymmetric synthesis and are often biologically active.^21^ The prochiral compound thioanisole was chosen as a model substrate (Scheme S3). The CHMOs exclusively produced the (*R*)‐sulfoxide, whereas TfPAMO produced both enantiomers, leading to an *ee* of only 16 % for the (*R*)‐sulfoxide (Figure S6B, Table S6).18c, 22

Having established that TmCHMO is an appealing biocatalyst based on its thermostability, solvent tolerance, and selectivity, we performed a more in‐depth characterization of its mechanistic properties. The reaction mechanism of a BVMO generally involves a C4a‐peroxyflavin intermediate that forms a tetrahedral Criegee intermediate through nucleophilic attack on the substrate carbonyl carbon (Scheme S4). Rearrangement of the Criegee intermediate yields the ester or lactone product.^23^ The spectral changes for TmCHMO during its catalytic cycle were monitored using a stopped‐flow spectrophotometer. Anaerobic reaction of TmCHMO with NADPH resulted in the loss of the absorbance peaks at 376 nm and 440 nm, which is consistent with the formation of the two‐electron‐reduced enzyme. After mixing the reduced TmCHMO with air‐saturated buffer, a rapid increase in absorbance at 355 nm was observed (*k*=37 s^−1^), together with a small absorbance decrease at 450 nm (Figure 2 A). These spectral changes are indicative of the formation of the C4a‐peroxyflavin intermediate. The absorbance at 355 nm was stable for 3 s and then slowly decreased (*k*=0.01 s^−1^) owing to decay of the intermediate, which is consistent with hydrogen peroxide elimination to form the reoxidized enzyme (*k*=0.004 s^−1^). In a second set of experiments, the anaerobically reduced TmCHMO was mixed with cyclohexanone in air‐saturated buffer. The absorbance at 355 nm increased for 0.1 s and then immediately decreased, which demonstrates the low kinetic stability of the intermediate in the presence of cyclohexanone (Figure 2 B and Figure S7). The rate of formation of the peroxyflavin was not influenced by the presence of cyclohexanone, while its decay rate was 80‐fold higher than that measured in the absence of this ketone. Collectively, these experiments suggest that TmCHMO functions as a typical BVMO, forming a stable flavin peroxide that can effectively perform substrate oxygenation. The kinetic stability of the peroxyflavin enables the enzyme to efficiently couple NADPH and dioxygen consumption with substrate oxygenation without leakage of hydrogen peroxide, which can be harmful in the context of large‐scale biotransformations.23a–23c

**Figure 2:**
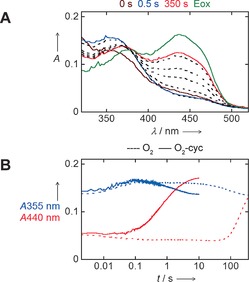
Reoxidation of TmCHMO. Reduced CHMO was reacted with air‐saturated buffer without (A, B) and with (B) cyclohexanone (cyc). Eox=spectrum of the completely oxidized CHMO.

For a more in‐depth understanding of TmCHMO properties, its crystal structure in complex with FAD and NADP^+^ in the oxidized and reduced states were solved to a resolution of 1.22 and 1.60 Å (Table S7; Figure 3 A, and Figure S8). In an attempt to rationalize the relatively high thermostability, we investigated the number of salt bridges present, since they are known to contribute to the thermostability of proteins.^24^ Our computational analysis of TfPAMO, TmCHMO, and RhCHMO identified 37, 31, and 16 salt bridges, respectively (Figure S9). This finding correlates with their *T*_m_ values (61, 48, and 37 °C, respectively). Mutagenesis on these BVMOs will be carried out to confirm the role of salt bridges in their stability.

**Figure 3:**
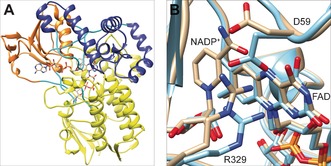
Overall structure of TmCHMO and its active site. A) Yellow: FAD domain; orange: NADPH domain; purple: helical domain; cyan: linker regions. B) Superposition of the active sites of the oxidized (orange) and reduced (cyan) forms.

The overall structures of the oxidized and reduced forms share an almost identical conformation, with a root mean square deviation of 0.20 Å for the backbone Cα atoms. However, inspection of the active sites shows distinct alterations (Figure 3 B and Figure S10). In the reduced enzyme, the electron density corresponding to the nicotinamide moiety of NADP^+^ is disordered, with no well‐defined electron density. Furthermore, R329 of the oxidized enzyme is engaged in H‐bonds with the carboxamide group of NADP^+^ and the side chain of D59. Upon enzyme reduction, R329 moves away from NADP^+^ and points toward the isoalloxazine moiety of the flavin ring, which favors an electrostatic interaction between the positively charged guanidinium group of R329 and the negatively charged reduced flavin. Upon formation of the flavin peroxide and concomitant loss of the negative charge on the flavin ring, R329 would shift back to the conformation interacting with the NADP^+^, thereby making the catalytic center accessible to a ketone substrate.23d These results confirm that the formation of the negatively charged reduced flavin is associated with a localized rearrangement of the central elements of the catalytic site. NADP^+^ is required for peroxyflavin formation and stabilization, primarily to provide essential H‐bonding interactions.

Inspecting the electron density of the oxidized structure of TmCHMO revealed that the putative substrate‐binding pocket is occupied by a ring‐shaped ligand (Figure 4 A and Figure S11). This region of electron density was putatively assigned to a molecule of nicotinamide, possibly resulting from the degradation of NADP^+^. Specifically, the ligand is placed right in front of the flavin ring and is in contact with residues T60, L145, L428, P430, F434, T435, and L437. This arrangement is very similar to that observed in the structures of RhCHMO_tight_ 23e and TfPAMO_MES_,23d both with a bound ligand (Figure 4 B and Figure S12). The ligand‐binding site is a compact cavity, which explains the general preference of TmCHMO for medium/small substrates.

**Figure 4:**
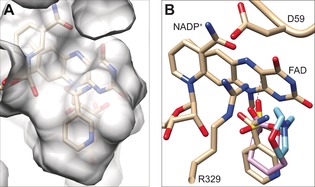
Ligand binding to oxidized TmCHMO. A) Semitransparent protein surface. B) Superposition of ligands in TmCHMO (pale orange carbon atoms), RhCHMO (PDB ID: 4RG3; pink carbon atoms), and TfPAMO (PDB ID: 2YLT; cyan carbon atoms).

To conclude, we report the discovery of a robust CHMO that shows great promise as an oxidative biocatalyst. The enzyme was found to be much more thermostable and solvent tolerant than known CHMOs. Furthermore, having established an effective recombinant production system and elucidated its crystal structure, TmCHMO provides the perfect starting point for engineering approaches to tune its properties.

## Supporting information

As a service to our authors and readers, this journal provides supporting information supplied by the authors. Such materials are peer reviewed and may be re‐organized for online delivery, but are not copy‐edited or typeset. Technical support issues arising from supporting information (other than missing files) should be addressed to the authors.

Supplementary
