# Supplementary material for: Characterization and Crystal Structure of a Robust Cyclohexanone Monooxygenase
Source: Angew Chem Int Ed Engl. 2016 Nov 22;55(51):15852–5. doi: 10.1002/anie.201608951 (PMC5213842; doi:10.1002/anie.201608951)
Supplement: Supplementary file 1 — Supplementary [file ANIE-55-15852-s001.pdf]

Angewandte Chemie
Eine Zeitschrift der Gesellschaft Deutscher Chemiker

Supporting Information

Characterization and Crystal Structure of a Robust Cyclohexanone Monooxygenase

Elvira Romero[+], J. Rubén Gómez Castellanos[+], Andrea Mattevi, and Marco W. Fraaije**

anie_201608951_sm_miscellaneous_information.pdf

Supporting Information

TABLE OF CONTENTS

1. Figures

2. Schemes

3. Tables

4. Experimental section

5. References

1. FIGURES

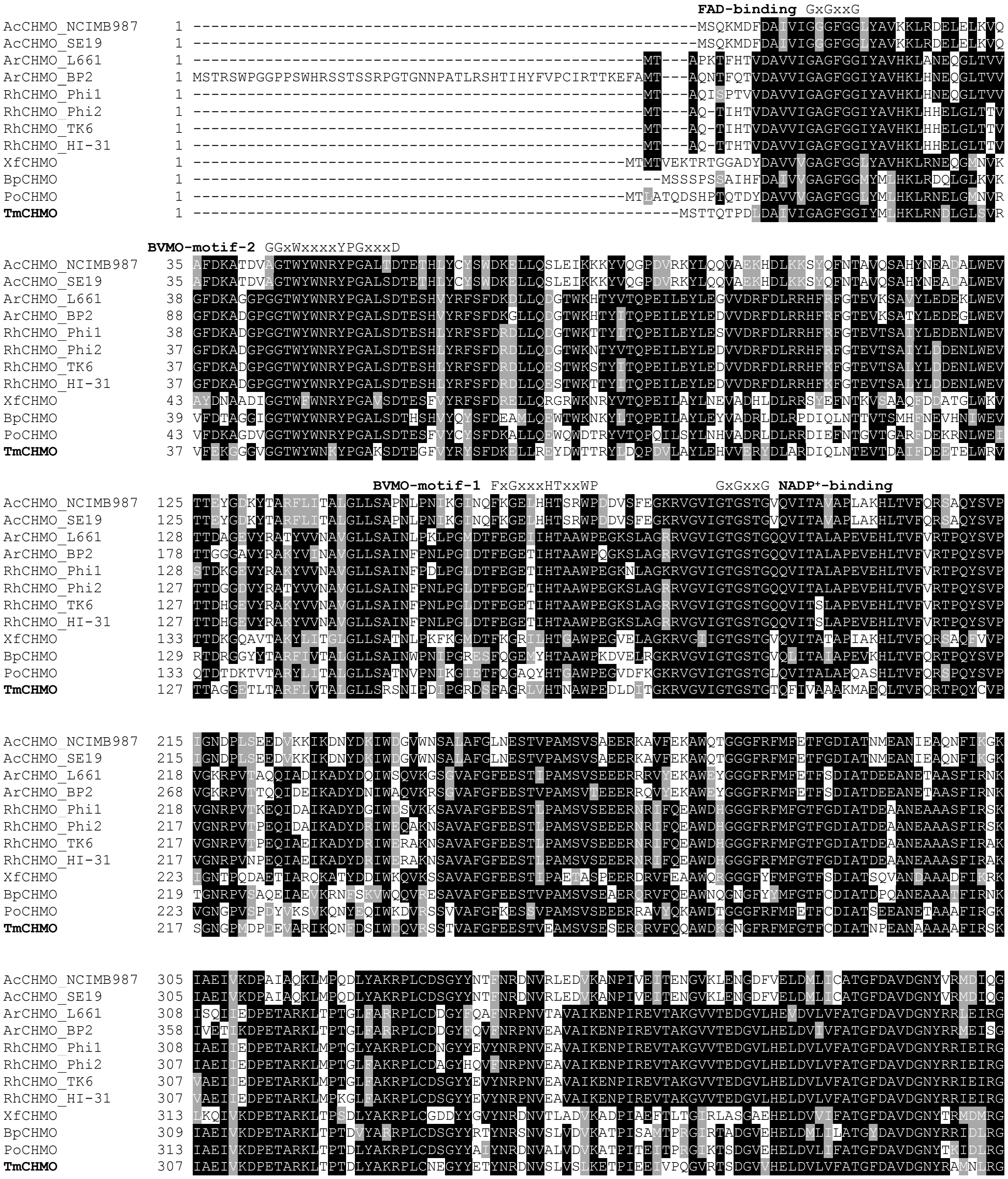

Figure S1. Protein sequence alignment of TmCHMO and other CHMOs. The amino acid sequences were retrieved from the NCBI database and their identification code is shown in Table S1. Clustal Omega program[1] was used to carry out the alignment and the BoxShade server (http://embnet.vital-it.ch/software/BOX_form.html) was used to edit it.

```
AcCHMO_NCIMB987  395 KNGLAMKDYWKEGPSSYMGVTVNNYPNMFMVLGPNGPFTNLPPSIESQVEWISDTIQYTVENNVESIEATKEAEEQWTQTCANIAEMTLF
AcCHMO_SE19      395 KNGLAMKDYWKEGPSSYMGVTVNNYPNMFMVLGPNGPFTNLPPSIESQVEWISDTIQYTVENNVESIEATKEAEEQWTQTCANIAEMTLF
ArCHMO_L661      398 RDGLNINDHWDGQPTSYLGVATANFPNWFMVLGPNGPFTNLPPSIETQVEWISATIDYAERNGIQAIEPTPEAEAEWTELCTQIANMTVF
ArCHMO_BP2       448 RDGVNINDHWDGQPTSYLGVSTAKFPNWFMVLGPNGPFTNLPPSIETQVEWISDTVAYAEENGIRAIEPTPEAEAEWTETCTQIANMTVF
RhCHMO_Phi1      398 RNGLHINDHWDGQPTSYLGVTTANFPNWFMVLGPNGPFTNLPPSIETQVEWISDTVAYAERNEIRAIEPTPEAEEEWTQTCTDIANATLF
RhCHMO_Phi2      397 RDGLHINDHWDGQPTSYLGVSTANFPNWFMVLGPNGPFTNLPPSIETQVEWISDTIGYAERNGVRAIEPTPEAEAEWTETCTAIANATLF
RhCHMO_TK6       397 RDGLHINDHWDGQPTSYLGVSTANFPNWFMVLGPNGPFTNLPPSIETQVEWISDTIGYAERNGVRAIEPTPEAEAEWTETCTEIANATLF
RhCHMO_HI-31     397 RDGLHINDHWDGQPTSYLGVSTANFPNWFMVLGPNGPFTNLPPSIETQVEWISDTIGYAERNGVRAIEPTPEAEAEWTETCTEIANATLF
XfCHMO           403 RNGVSLRDMWKEGPLGYLGIMEAEFPNLFMILGPNGPFTNLPPSIETQVEWIADMVKTMEAKGLKTSEPTAQARDQWVELCRTIANMTLF
BpCHMO           399 RGGQTINEHWNDTPTSYVGVSTANFPNMFMILGPNGPFTNLPPSIEAQVEWITDLVAHMRQHGLATAEPTRDAEDAWGRTCAEIAEQTLF
PoCHMO           403 RNGKTIKDKWKAGPTSYLGVASADYPNLFMILGPNGPFTNLPPSIETQVEWISDLIKHMNDTGRQLVEATHEGEDGWTATCNEIAGYTLF
TmCHMO           397 RDGRHINEHWTEGPTSYLGVTKAGFPNMFMILGPNGPFTNLPPSIEAQVEWISDLIDKATREGLTTVEPTADAEREWTETCAEIANMTLF

AcCHMO_NCIMB987  485 PKAQSWIFGANIPGKKNTVYFYLGGLKEYRSALANCKNHAYEGFDIQLQRSDIKQPANA
AcCHMO_SE19      485 PKAQSWIFGANIPGKKNTVYFYLGGLKEYRSALANCKNHAYEGFDIQLQRSDIKQPANA
ArCHMO_L661      488 TKVESWIFGANVPGKKPSVLFYLGGLSNYRGVLDDVTANGYRGFELKSEAPVAA-----
ArCHMO_BP2       538 TKVDSWIFGANVPGKKPSVLFYLGGLGNYRGVLDDVTANGYRGFELKSEAAVAA-----
RhCHMO_Phi1      488 TRGDSWIFGANVPGKKPSVLFYLGGLGNYRNVLAGVVADSYRGFELKSAVPVTA-----
RhCHMO_Phi2      487 TKGDSWIFGANIPGKTPSVLFYLGGLRNYRAVLAEVATDGYRGFDVKSAEMVTV-----
RhCHMO_TK6       487 TKGDSWIFGANIPGKKPSVLFYLGGLRNYRAVMAEVAADGYRGFEVKSAEMVTV-----
RhCHMO_HI-31     487 TKGDSWIFGANIPGKKPSVLFYLGGLRNYRAVMAEVAADGYRGFEVKSAEMVTV-----
XfCHMO           493 PKAESWIFGANIPGKKNTVMFYLAGLGNYRKVLSGLSESGYPTIIFDRAVECVA-----
BpCHMO           489 GQVESWIFGANSPGKKHTLMFYLAGLGNYRKQLADVANAQYQGFAFQPL----------
PoCHMO           493 PKADSWIFGANIPGKARTVMFYLAGLGAYTQKLNEVTSTGYPGFEIR------------
TmCHMO           487 PKADSWIFGANIPGKRHAVMFYLGGLGNYRRQLADVADGGYRGFQLRGERAQAVA----
```

Figure S1. (Continued).

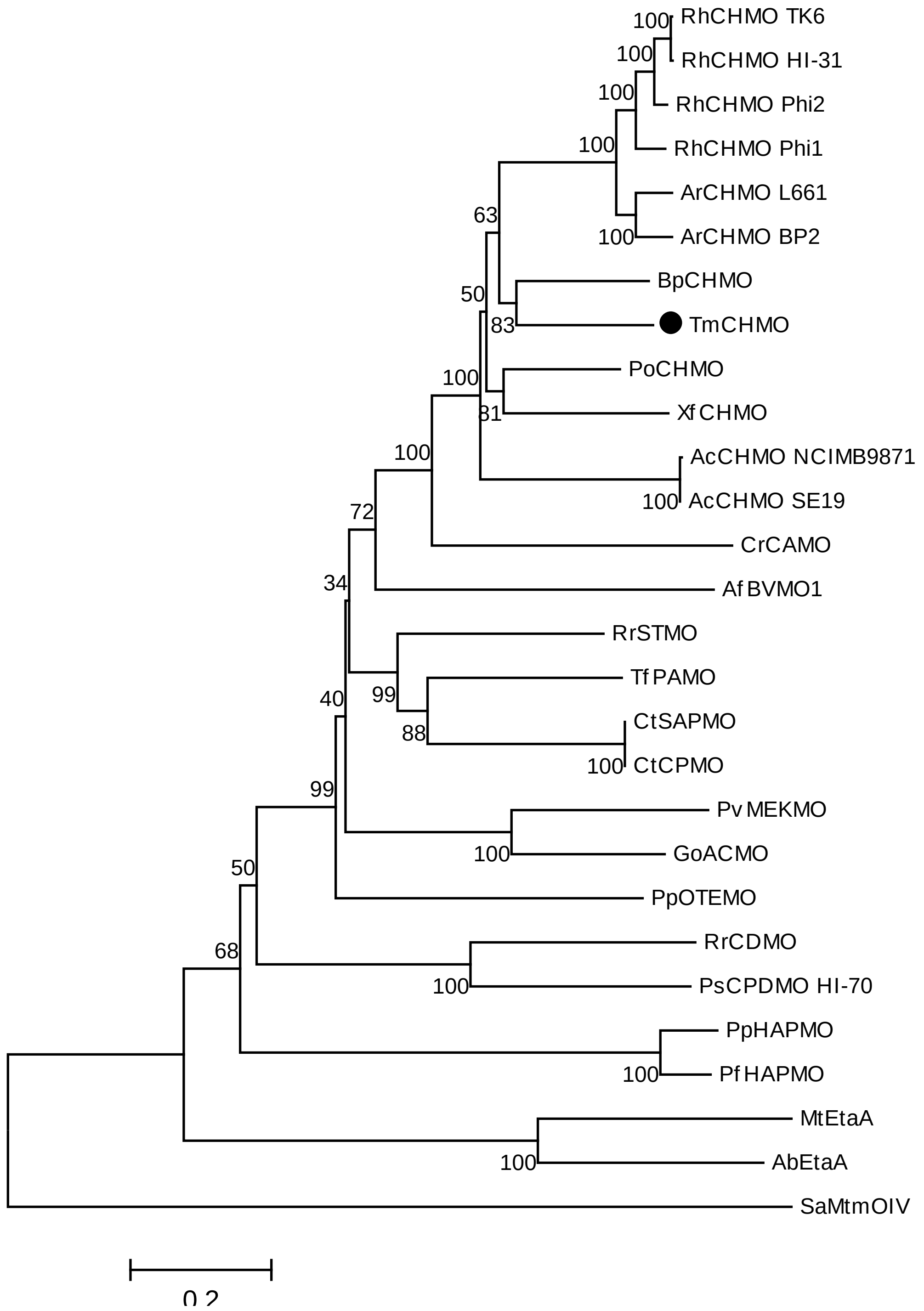

Figure S2. Phylogenetic analysis of TmCHMO (●) and other BVMOs. The amino acid sequences were retrieved from the NCBI database and their identification code is shown in Table S1. A ClustalW alignment of these sequences was used to construct the phylogenetic tree using the Neighbor-joining method.[2] Bootstrap values are shown next to the branches (100 replicates). These analyses were carried out using the MEGA7 software.[3]

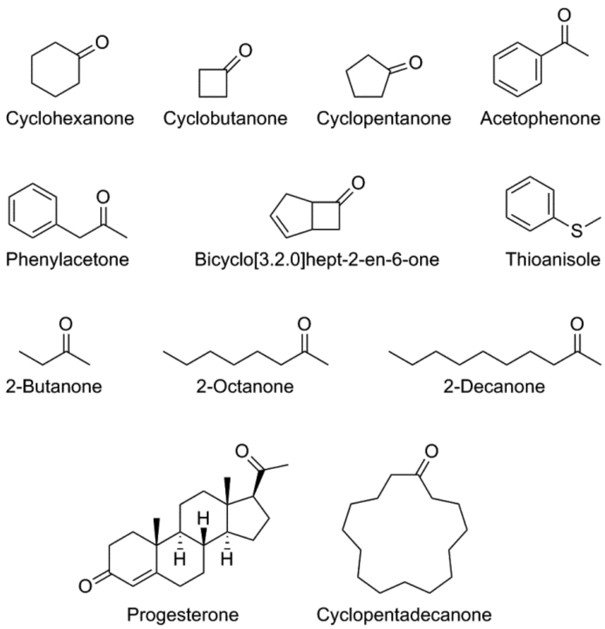

Figure S3. Compounds assayed as a substrate for TmCHMO and AcCHMO.

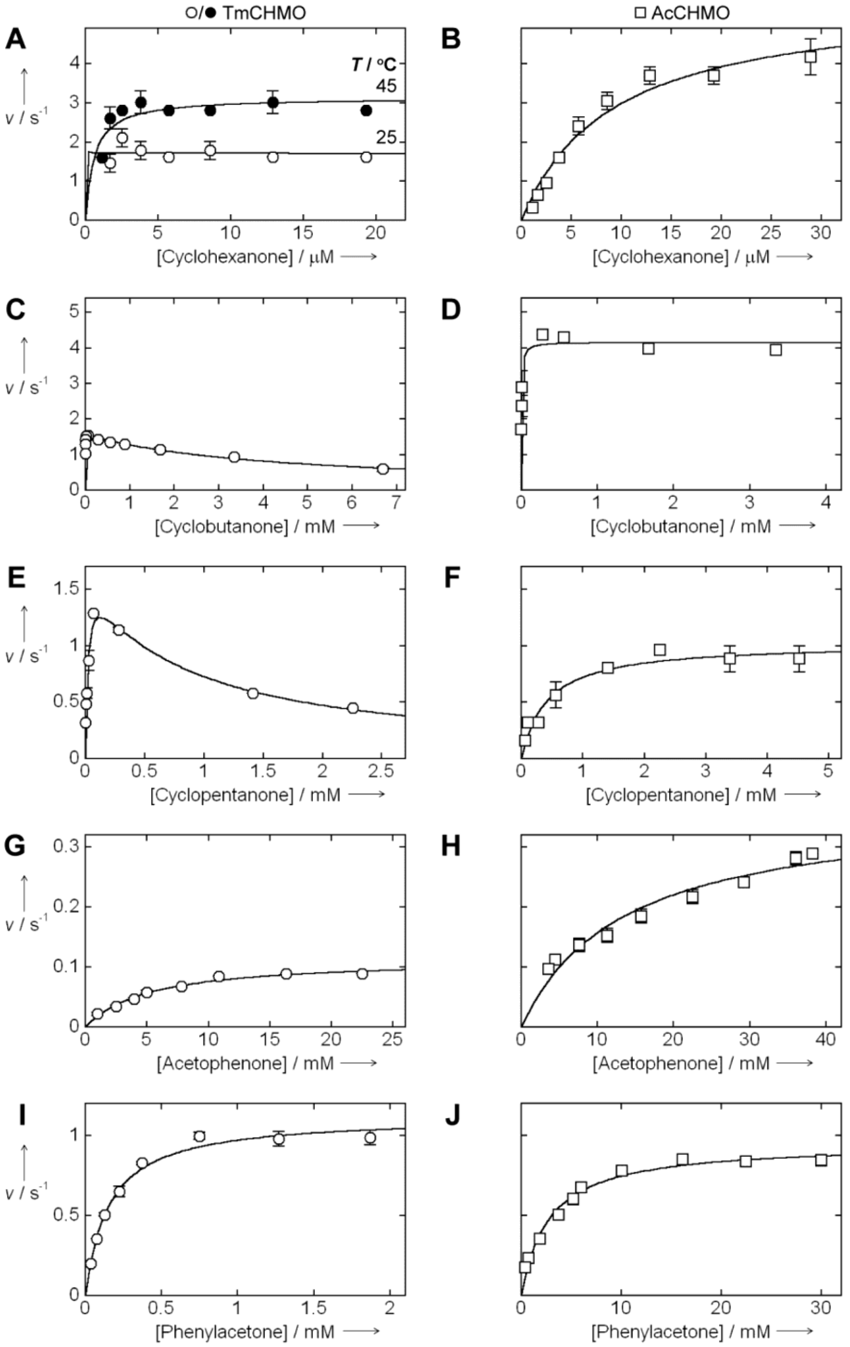

Figure S4. NADPH consumption rates for TmCHMO and AcCHMO at increasing substrate concentrations. Enzyme (0.02-4 μM), NADPH (150 μM), and substrate were reacted in air-saturated 50 mM Tris-HCl at pH 7.0 and 25 °C. These plots were fit to either the Michaelis-Menten equation or the substrate inhibition equation to determine the steady-state kinetic parameters shown in Table S3.

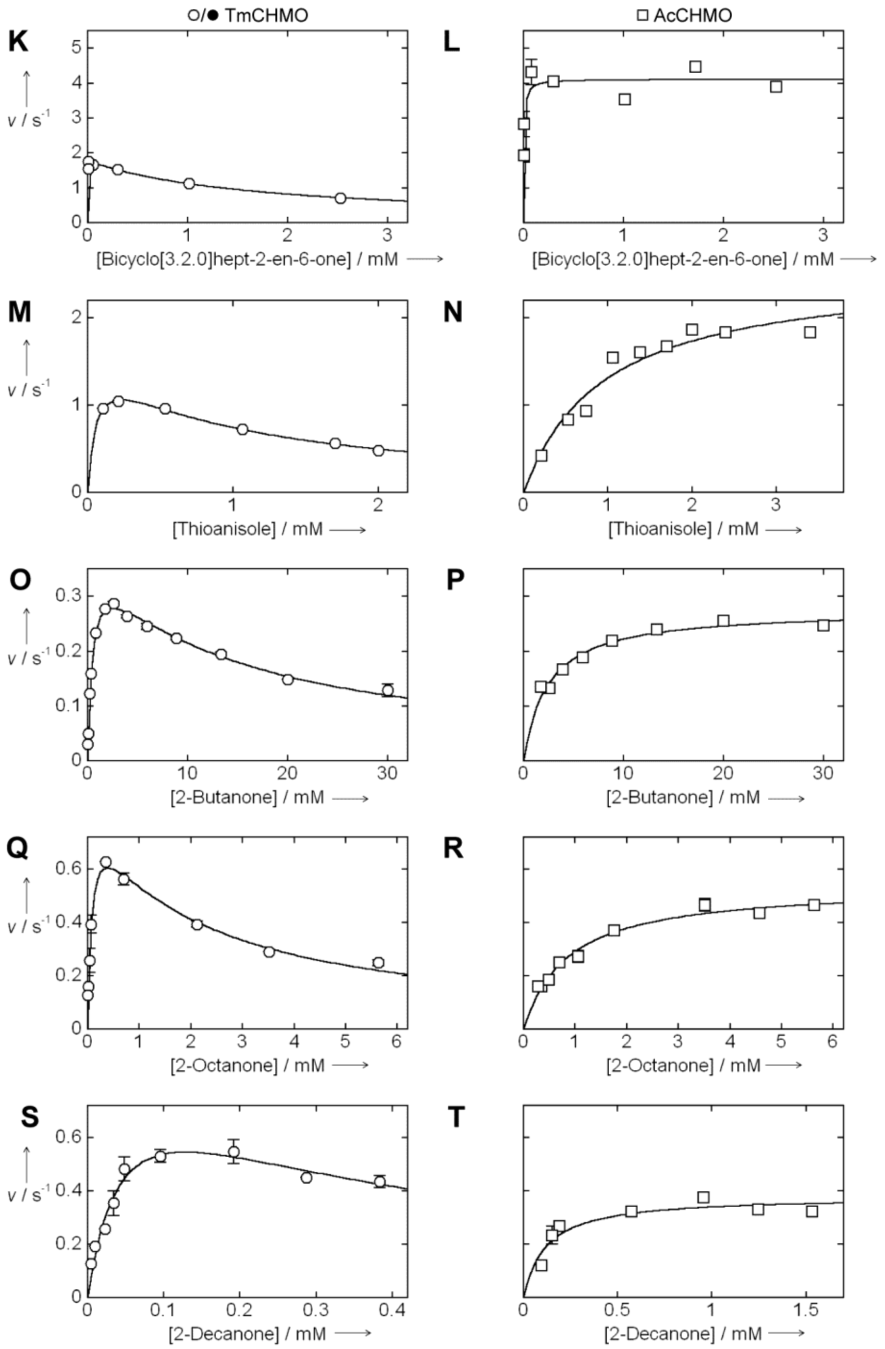

Figure S4. (Continued).

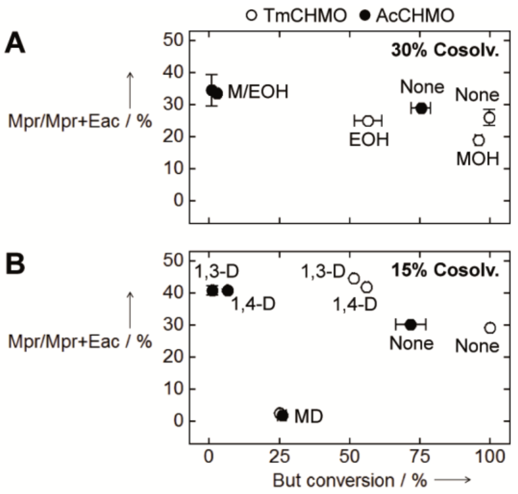

Figure S5. Effect of cosolvents on the conversion yield and regioselectivity in the reaction of TmCHMO and AcCHMO with 2-butanone. Reactions contained purified enzyme (1 μM), 2-butanone (11 mM), NADPH (150 μM), and phosphite (20 mM) in 50 mM Tris-HCl at pH 7.0 with or without a cosolvent. Concentration of 2-butanone (but), methyl propanoate (mpr) and ethyl acetate (eac) was determined by headspace GC-MS after 48 h at 17 °C. MOH, methanol; EOH, ethanol; 1,3-D, 1,3-dioxane; 1,4-D, 1,4-dioxane; and MD, 2-methyl-1,3-dioxolane.

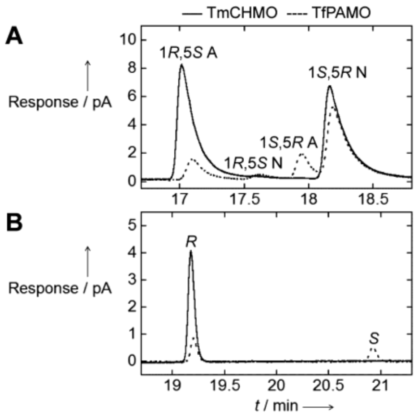

Figure S6. Products of the reaction of TmCHMO and TfPAMO with *rac*-bicyclo[3.2.0]hept-2-en-6-one (A) and thioanisole (B). Reactions contained purified enzyme (2 μM), *rac*-bicyclo[3.2.0]hept-2-en-6-one (11 mM) or thioanisole (3.4 mM), NADPH (150 μM), and phosphite (20 mM) in 50 mM Tris-HCl at pH 7.0. Reactions containing *rac*-bicyclo[3.2.0]hept-2-en-6-one and thioanisole were analysed by chiral GC after 20 h and 2 h at 24 °C, respectively. Both enantiomers of bicyclo[3.2.0]hept-2-en-6-one were fully converted by CHMO yielding almost exclusively one regioisomer from each enantiomer, namely (1*S*,5*R*)-2-oxabicyclo[3.3.0]oct-6-en-3-one (normal product) and (1*R*,5*S*)-3-oxabicyclo[3.3.0]oct-6-en-2-one (abnormal product).

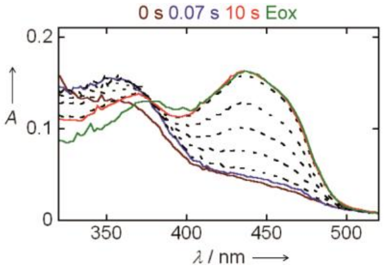

Figure S7. Oxidative half-reaction for TmCHMO. Spectral changes observed during the reaction of reduced enzyme (12 µM) with cyclohexanone (100 µM) in air-saturated 50 mM Tris-HCl at pH 7.0 and 25 °C. Spectra obtained after 0.001, 0.07, 0.1, 0.3, 0.6, 1, 5, and 10 s are shown. The spectrum of the oxidized enzyme, before mixing with NADPH (19.2 µM), is shown (Eox).

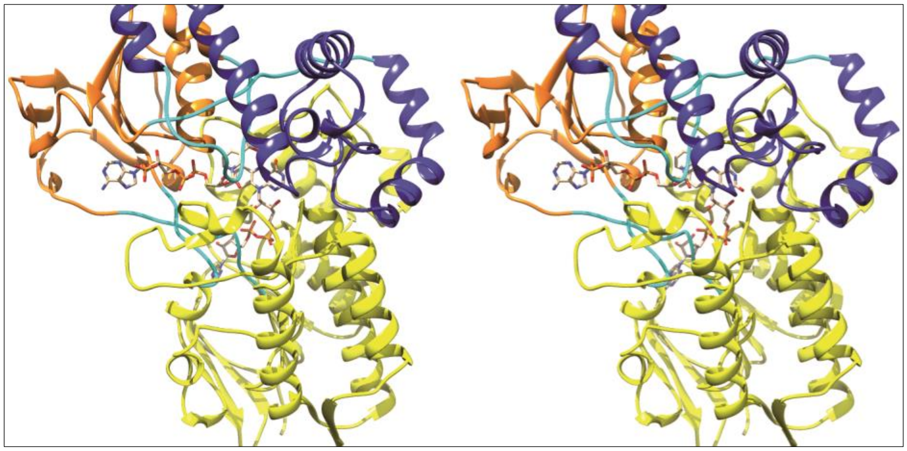

Figure S8. Stereo view of the crystal structure of TmCHMO. Yellow, FAD domain (residues 1-141 and 387-540); orange, NADPH domain (152-210 and 334-380); purple, helical domain; cyan, linker regions (224-320).

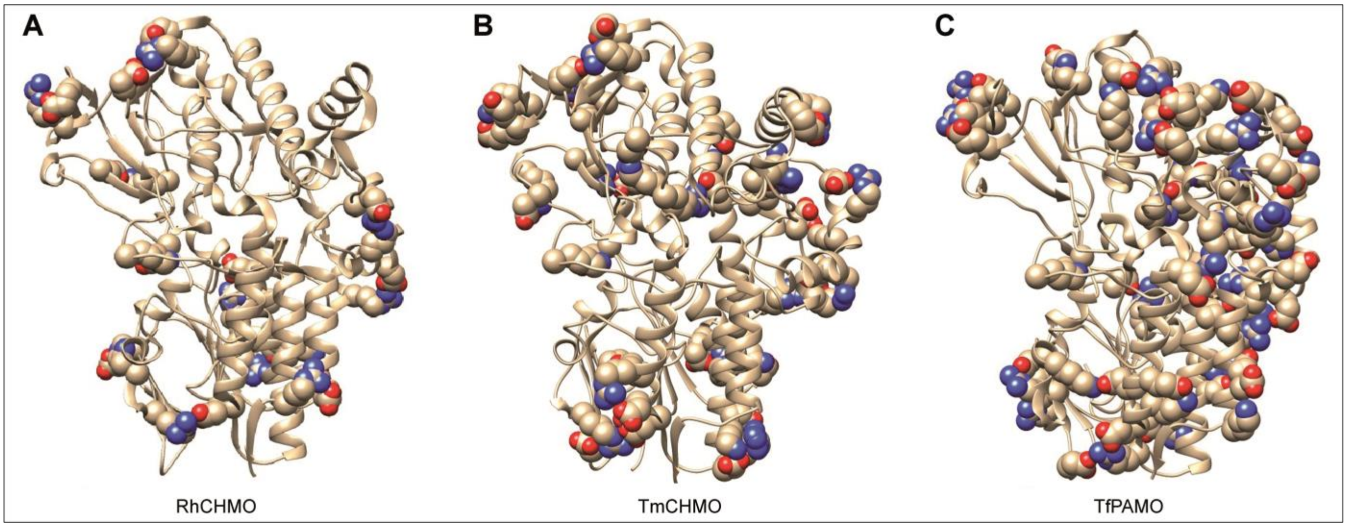

Figure S9. Crystal structures of BVMOs highlighting the salt bonds (red and blue atoms). Using the salt bridges plug-in for VMD,[4] 16, 31 and 37 salt bridges were measured for RhCHMO (PDB: 4RG3; A), TmCHMO (B) and TfPAMO (PDB: 2YLT; C), respectively, which indicates a correlation between the amount of salt bridges and the thermostability of a BVMO. Figure made with Chimera.[5]

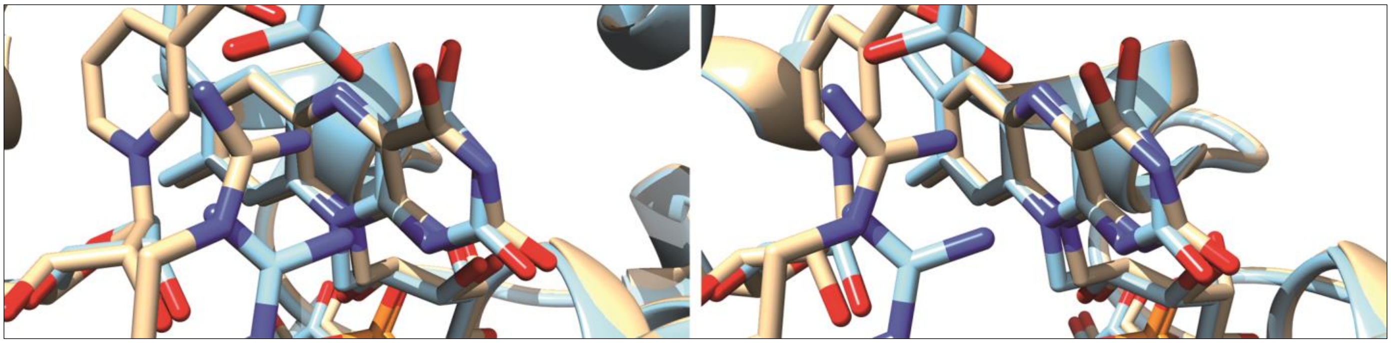

Figure S10. Stereo view of the superimposed active sites of the oxidized (orange) and reduced (cyan) TmCHMO crystal structures.

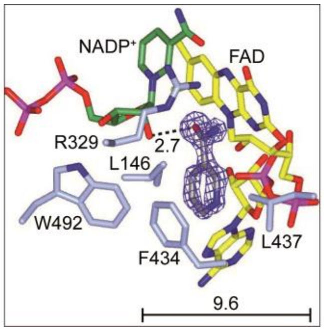

Figure S11. $2F_o$-F_c electron density map (1.6 σ level) of putative nicotinamide ligand bound to the oxidized TmCHMO. The map was calculated before inclusion of the ligand in the refinement. Distances are indicated in Å.

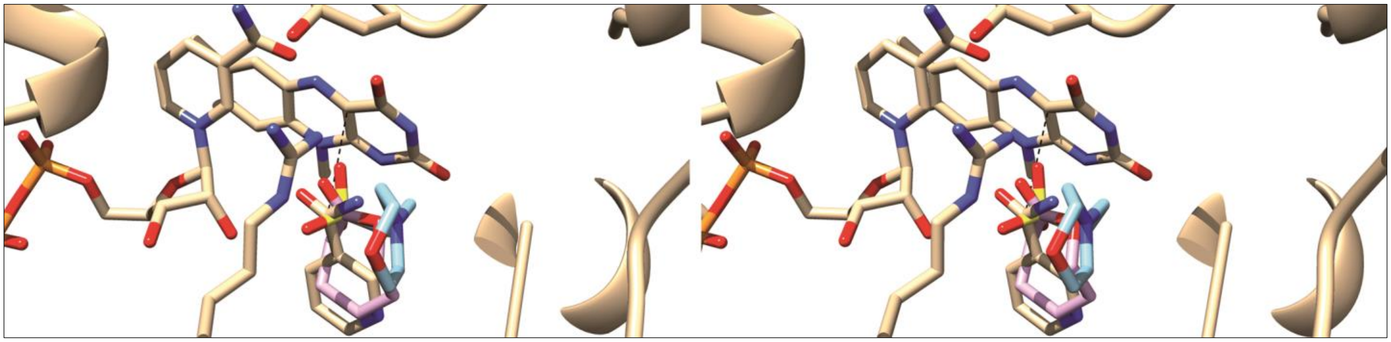

Figure S12. Stereo view of the superimposed ligands in TmCHMO (orange carbons), RhCHMO (PDB: 4RG3; pink carbons) and TfPAMO (PDB: 2YLT; cyan carbons).

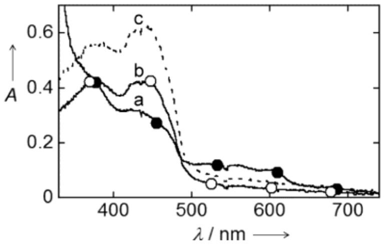

Figure S13. Microspectrophotometry of TmCHMO crystals measured at 100 K. Soaking the crystals at room temperature prior to cryo-cooling in dithionite solutions leads to reduction of the crystalline enzyme (a). Reduced crystals can be reoxidized by soaking at room temperature in aerated solutions as shown by spectra b and c, which were collected on crystals that were cryo-cooled 20 s and 40 s after beginning of reoxidation, respectively. Spectrum c corresponds to that of the fully reoxidized enzyme, which is indistinguishable from that measured on oxidized crystals directly harvested from the crystallization droplets.

2. SCHEMES

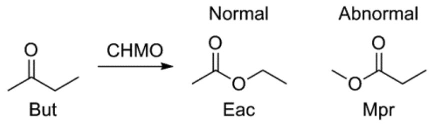

Scheme S1. Enzyme-catalyzed Baeyer-Villiger oxidation of 2-butanone (but) yielding ethyl acetate (eac) and methyl propanoate (mpr).

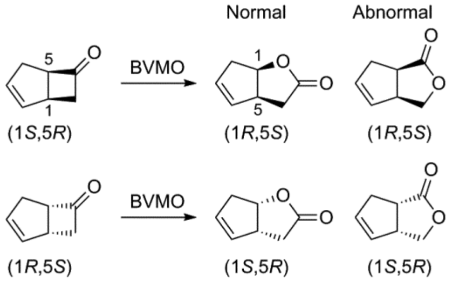

Scheme S2. Enzyme-catalyzed Baeyer-Villiger oxidation of *rac*-bicyclo[3.2.0]hept-2-en-6-one.

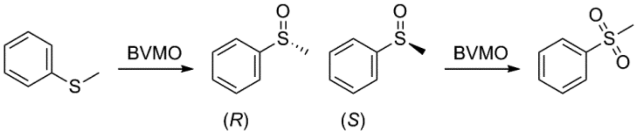

Scheme S3. Enzyme-catalyzed sulfoxidation of thioanisole (methyl phenyl sulfide) to (*R*/*S*)-methyl phenyl sulfoxide and methyl phenyl sulfone.

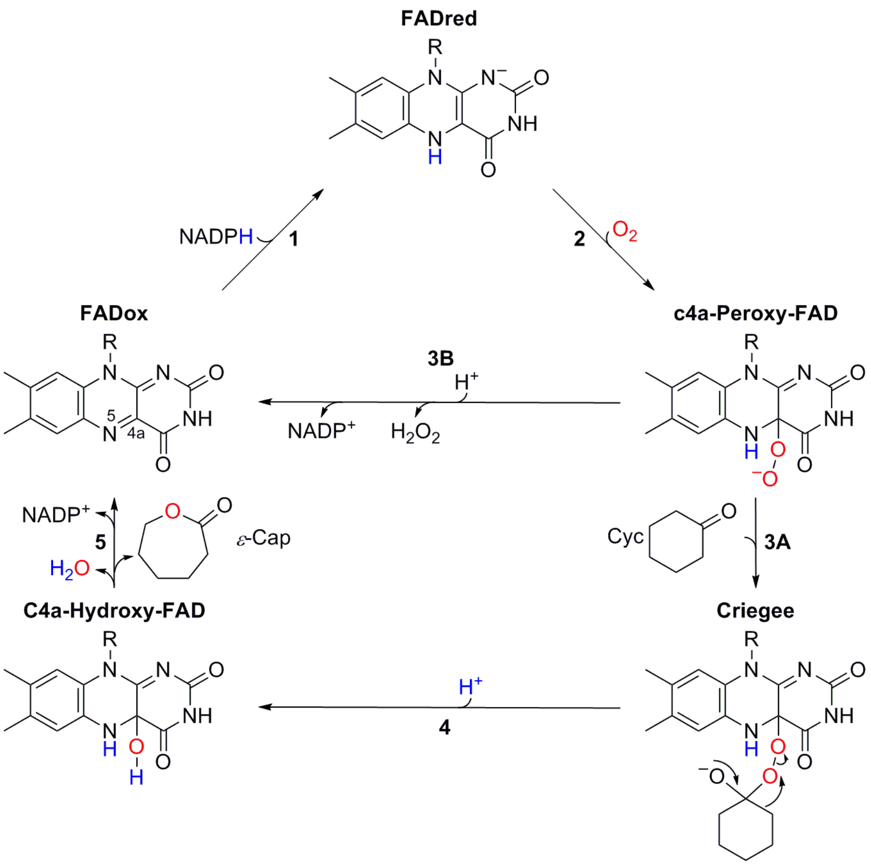

Scheme S4. Catalytic cycle of TmCHMO with cyclohexanone (Cyc) as a substrate. For FAD, *R* is ribitol adenosine diphosphate. *ε*-Caprolactone (*ε*-Cap) is produced.

3. TABLES

Table S1. BVMOs included in Figure S1 and S2.

BVMO	Sources	Name	NCBI ID
2-Oxo-Δ(3)-4,5,5-trimethylcyclopentenylacetyl-CoA monooxygenase	*Pseudomonas putida* ATCC	PpOTEMO	WP_032492627.1
4-Hydroxyacetophenone monooxygenase	*Pseudomonas fluorescens* ACB	PfHAPMO	AAK54073.1
	Pseudomonas putida JD1	PpHAPMO	ACJ37423.1
4-Sulfoacetophenone monooxygenase	*Comamonas testosteroni* KF-1	CtSAPMO	EED70489.1
Acetone monooxygenase	*Gordonia* sp. TY-5	GoACMO	BAF43791.1
Baeyer-Villiger monooxygenase Af1	*Aspergillus fumigatus* Af293	AfBVMO1	XP_747160
Cycloalkanone monooxygenase	*Cylindrocarpon radicicola* ATCC 11011	CrCAMO	AET80001.1
Cyclododecanone monooxygenase	*Rhodococcus ruber*	RrCDMO	AAL14233.1
Cyclohexanone monooxygenase	*Acinetobacter calcoaceticus* NCIMB9871	AcCHMO NCIMB9871	BAA86293.1
	Acinetobacter sp. SE19	AcCHMO SE19	AAG10021.1
	Arthrobacter sp. L661	ArCHMO L661	ABQ10653.1
	Arthrobacter sp. BP2	ArCHMO BP2	AAN37479.1
	Brachymonas petroleovorans	BpCHMO	AAR99068.1
	Polaromonas sp. JS666	PoCHMO	WP_011486392.1
	Rhodococcus sp. HI-31	RhCHMO HI-31	BAH56677.1
	Rhodococcus sp. TK6	RhCHMO TK6	AAR27824.1
	Rhodococcus sp. Phi2	RhCHMO Phi2	AAN37491.1
	Rhodococcus sp. Phi1	RhCHMO Phi1	AAN37494.1
	Thermocrispum municipale DSM 44069	TmCHMO	WP_028849141.1
	Xanthobacter flavus	XfCHMO	CAD10801.1
Cyclopentadecanone monooxygenase	*Pseudomonas* sp. HI-70	PsCPDMO	BAN84077.1
Cyclopentanone monooxygenase	*Comamonas testosteroni*	CtCPMO	CAD10798.1
Ethionamide monooxygenase	*Acinetobacter baumannii*	AbEtaA	EXB35767.1
	Mycobacterium tuberculosis	MtEtaA	P9WNF9.1
Methylethylketone monooxygenase	*Pseudomonas veronii* MEK700	PvMEKMO	ABI15711.1
Mithramycin monooxygenase	*Streptomyces argillaceus*	SaMtmOIV	CAK50794.2
Phenylacetone monooxygenase	*Thermobifida fusca*	TfPAMO	WP_011291921.1
Steroid Monooxygenase	*Rhodococcus rhodochrous*	RrSTMO	BAA24454.1

Table S2. Enzymes involved in the cyclohexanol degradation pathway in *A. calcoaceticus* NCIMB9871 and proposed homologues in *T. municipale*.[a]

A. calcoaceticus NCIMB9871	*T. municipale*	Sequence identity [%]
6-Hydroxyhexanoate dehydrogenase (BAC80217.1; chnD)	Alcohol dehydrogenase (WP_028849137.1; CDS: 212868-213932)	39
6-Oxohexanoate dehydrogenase (BAA86294.1; chnE)	NAD-dependent succinate-semialdehyde dehydrogenase (WP_028849138.1; CDS: 214085-215431)	32
6-Hexanolactone hydrolase (BAC80218.1; chnC)	6-Hexanolactone hydrolase (WP_043659041.1; CDS: 217744-218673)	60
CHMO (BAA86293.1; chnB)	CHMO (WP_028849141.1; CDS: 218708-220333)	57

[a] The amino acid sequence of the above *A. calcoaceticus* proteins was aligned with the sequence of the translated genes placed down- and upstream of the TmCHMO gene. Putative *T. municipale* homologues to *A. calcoaceticus* chnC, chnD, and chnE are proposed based on the sequence identity and the proximity to the TmCHMO gene.

Table S3. Steady-state kinetic parameters for AcCHMO and TmCHMO.[a]

	TmCHMO			AcCHMO		
Substrate	k_{cat}	K_m	k_{cat}/K_m	k_{cat}	K_m	k_{cat}/K_m
Cyclohexanone	2	<0.001	≥2,000	6	0.009	667
Cyclobutanone	2	<0.001	≥2,000	4	<0.004	≥1,000
Cyclopentanone	2	0.02	100	1	0.4	3
Acetophenone	0.1	5	0.02	0.4	13	0.03
Phenylacetone	1	0.2	5	1	3	0.3
Bicyclo[3.2.0]hept-2-en-6-one	2	<0.001	≥2,000	4	<0.005	≥800
Thioanisole	2	<0.06	≥33	3	1	3
2-Butanone	0.4	0.5	1	0.3	<2	≥0.2
2-Octanone	1	0.1	10	1	1	1
2-Decanone	1	0.05	20	0.4	<0.1	≥4

[a] Reactions contained enzyme (0.02-4 μM), NADPH (150 μM), and increasing substrate concentrations in air-saturated 50 mM Tris-HCl at pH 7.0 and 25 °C. k_{cat} [s^{-1}], K_m [mM], and k_{cat}/K_m [$mM^{-1}s^{-1}$] were determined as described in Figure S4. The K_m value was assumed to be <0.001 mM with certain substrates for either CHMO, since an accurate K_m value could not be determined due to the very high enzyme affinity.

Table S4. Melting temperature for CHMOs.[a]

Additive	TmCHMO	AcCHMO
0 mM NAD(P)$^+$	48	37
0.5 mM NADP$^+$	51	40
0.5 mM NAD$^+$	49	37

[a] Samples contained 10 μM CHMO in 50 mM Tris-HCl at pH 7.0. The standard deviation is 1≤% and based on two replicates.

Table S5. Effect of cosolvents on the reaction of CHMO with 2-butanone and characteristics of cosolvents.[a]

Cosolvent	Structure	*ε*[b]	*I*[c]	Log*P*[d]	DC[e]	Cosolvent [%]	But conversion [%][f]		Mpr/Mpr+Eac [%]	
							TmCHMO	AcCHMO	TmCHMO	AcCHMO
None						0	100	75	26	29
Methanol	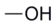	32.7	5.1	-0.8	30.5	30	96	3	19	34
Ethanol	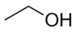	24.6	4.3	-0.2	54.4	30	56	1	25	35
1,4-Dioxane	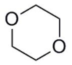	2.2	4.8	-1.1	92.1	15	56	7	42	41
1,3-Dioxane	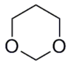					15	51	1	45	41
2-Methyl-1,3-dioxolane	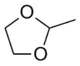					15	25	26	3	2

[a] Reactions were analyzed after 48 h at 17 ºC. The standard deviation bars are shown in Figure S5. Characteristics of cosolvents were obtained from.[6] [b] *ε*, solvent dielectric constant. [c] *I*, polarity index. [d] *P*, partition coefficient using 1-octanol:water as a reference system. [e] DC, denaturation capacity. [f] Conversion of 2-butanone (but) yields ethyl acetate (eac) and methyl propanoate (mpr).

Table S6. Conversion of *rac*-bicyclo[3.2.0]hept-2-en-6-one and thioanisole using purified BVMOs.[a]

BVMO	*rac*-Bicyclo[3.2.0]hept-2-en-6-one				Thioanisole	
	c [%][b]	N:A [%][c]	ee_N [%][d]	ee_A [%]	*c* [%]	*ee* [%]
TmCHMO	100	49:51	99 (1*S*,5*R*)	100 (1*R*,5*S*)	100	100 (*R*)
AcCHMO	100	49:51	97 (1*S*,5*R*)	100 (1*R*,5*S*)	100	100 (*R*)
TfPAMO	65	71:29	91 (1*S*,5*R*)	1 (1*S*,5*R*)	48	16 (*R*)

[a] Reactions were analysed after 20 h (*rac*-bicyclo[3.2.0]hept-2-en-6-one) and 2 h (thioanisole) at 24 ºC. [b] *c*, extent of conversion of substrate (*S*); $c = [1 - ([S]_f/[S]_0)] \times 100$. [c] N, normal product; A, abnormal product. [d] *ee*, enantiomeric excess of the product; $ee = [(A_{major} - A_{minor})/(A_{major} + A_{minor})\ ] \times 100$ where A_{major} and A_{minor} are the areas obtained by chiral GC for the major and minor enantiomers, respectively. Major enantiomer is indicated in parenthesis.

Table S7. Data collection and refinement statistics.[a]

	Oxidized enzyme<br>PDB: 5M0Z	Reduced enzyme<br>PDB: 5M10
Wavelength (Å)	1.0	0.9686
Resolution range	38.9-1.22 (1.26-1.22)	33.8-1.6 (1.66-1.6)
Space group	$C222_1$	$C222_1$
Unit cell (Å), (°)	68.84 113.63 155.48, 90 90 90	69.21 114.68 155.43, 90 90 90
Total reflections	309305 (28806)	146832 (15162)
Unique reflections	168622 (15498)	76543 (7786)
Multiplicity	1.8 (1.9)	1.9 (1.9)
Completeness (%)	93 (087)	94 (97)
Mean I/sigma (I)	8.36 (1.21)	12.23 (1.81)
Wilson B-factor ($Å^2$)	9.86	10.11
R-merge (%)	4.1 (55.1)	6.4 (43.4)
CC1/2	0.999 (0.701)	0.996 (0.664)
Reflections used in refinement	168273 (15418)	76541 (7786)
Reflections used for R-free	5106 (424)	2340 (237)
R-work (%)	14.38 (26.63)	14.79 (26.55)
R-free	17.48 (32.14)	17.74 (28.44)
Number of non-hydrogen atoms	5034	5096
Macromolecule	4315	4185
Ligands	156	104
Protein residues	529	529
RMS (bonds) (Å)	0.613	0.618
RMS (angles) (°)	5.13	4.76
Ramachandran favoured (%)	98	98
Ramachandran allowed (%)	1.8	1.7
Ramachandran outliers (%)	0.18	0.38
Rotamer outliers (%)	0.87	0.91
Clashscore	4.21	4.29
Average B-factor	16.35	13.69
Macromolecules	14.81	11.46
Ligands	16.20	8.10
Solvent	28.21	25.93

[a] Statistics for the highest-resolution shell are shown in parentheses. Data were obtained with PHENIX.[7]

4. EXPERIMENTAL SECTION

Materials. NADPH was acquired from Jülich Chiral Solutions GmbH. All other materials were acquired from Sigma-Aldrich unless otherwise specified.

Cloning of TmCHMO and BVMOs Purification. The amino acid sequence of a putative CHMO from the thermophilic actinomycete *T. municipale* DSM 44069 was retrieved from the NCBI database (http://www.ncbi.nlm.nih.gov). This organism is able to grow at 28-60 °C.[8] The TmCHMO sequence was found by carrying out a BLASTP (http://blast.ncbi.nlm.nih.gov/Blast.cgi) using the amino acid sequence of AcCHMO as a query sequence. A codon-optimized TmCHMO gene was synthetized and cloned into pUC57 plasmid by GenScript. The TmCHMO gene was subcloned using standard methods into the pCRE2 plamid[9] with XhoI and HindIII restriction sites at the 5' end and 3' end, respectively. The resulting plasmid is a pBAD vector harboring a gene encoding TmCHMO fused to the C-terminus of a thermostable variant of phosphite dehydrogenase (PTDH). A histidine tag at the N-terminus of the PTDH is also included in this construct. The same construct containing either the AcCHMO gene or the TfPAMO gene, instead of the TmCHMO gene, was carried out for a previous study[9] and used here to obtain the corresponding fusion proteins. The TmCHMO gene was also subcloned into a pBAD plasmid containing the SUMO (small ubiquitin-related modifier) gene with a N-terminal histidine tag (pBAD-His-Tag-SUMO-TmCHMO).[10] In this construct, the SUMO gene is at the N-terminus of the TmCHMO gene. NdeI and HindIII restriction sites are placed at the 5' end and 3' end of the TmCHMO gene, respectively. All enzymes were expressed using NEB 10-β *Escherichia coli* cells in the presence of L-arabinose and purified using Ni-sepharose resin, as previously described.[11] The purified HisTag-SUMO-TmCHMO fusion protein was incubated overnight with SUMO protease. Subsequently, a Ni^{2+}-Sepharose column was used to capture the SUMO-His-Tag protein yielding isolated TmCHMO in the flow through.

Phylogenetic Analyses. The evolutionary relationships between TmCHMO and 27 BVMOs were analyzed using the MEGA7 software.[3] The amino acid sequences used for this analysis were retrieved from the NCBI database and their identification code is shown in Table S1. A ClustalW alignment of these sequences was used to construct a Neighbor-joining phylogenetic tree.[2] The percentage of replicate trees in which the associated taxa clustered together in the bootstrap test (100 replicates)[12] was determined. The Poisson correction method[13] was used to calculate the evolutionary distances.

Determination of the Extinction Coefficient. For all experiments described in this work, purified enzyme concentration was determined based on the corresponding extinction coefficient. The extinction coefficient of WT AcCHMO ($\varepsilon_{440\ nm}$ = 13.8 mM^{-1} cm^{-1}) and TfPAMO ($\varepsilon_{441\ nm}$ = 12.4 mM^{-1} cm^{-1}) was obtained from previously published studies.[14] The extinction coefficient of TmCHMO was determined to be 14.0 mM^{-1} cm^{-1}, in 50 mM Tris-HCl at pH 7.0. It was calculated as previously described for AcCHMO,[14a] except that the FAD was released from the enzyme by heat denaturation (30 min, 100 °C). Non-covalently bound FAD incorporation for TmCHMO was determined to be 85% by comparing the protein concentration measured using the Bradford assay (BioRad) to the concentration measured based on the extinction coefficient at 440 nm.

Steady-State Kinetics. Steady-state kinetic parameters for purified TmCHMO and AcCHMO were determined using a V-660 spectrophotometer (Jasco). Enzyme (0.02-4 μM), NADPH (150 μM), and increasing substrate concentrations were reacted in air-saturated 50 mM Tris-HCl at pH 7.0 and 25 °C. The rate of NADPH consumption was determined by following the decrease in absorbance at 340 nm (ε_{340} = 6.22 mM^{-1} cm^{-1}). The initial rates of the reactions were plotted as a function of the substrate concentrations. These data were fit to either the Michaelis-Menten equation or the substrate inhibition equation using the KaleidaGraph software (Synergy Software, Reading, PA). Among the assayed compounds (Figure S3), only progesterone and cyclopentadecanone (50 μM) were not substrates of either enzyme based on the NADPH consumption assay.

Rapid Kinetics. The reoxidation of TmCHMO was studied using a SX20 stopped-flow spectrometer in double-mixing mode. A xenon lamp and a photodiode array (PDA) detector were used. The enzyme was

first anaerobically reduced with 1.6 equivalents of NADPH in the aging loop of the stopped-flow apparatus for 10 s. The anaerobically reduced enzyme was subsequently mixed with air-saturated buffer in the absence or the presence of cyclohexanone. The final concentration of enzyme and cyclohexanone was 12 and 100 µM, respectively. All solutions were prepared in 50 mM Tris-HCl at pH 7.0. All reactions were run at 25 °C in duplicate or triplicate by mixing equal volumes of reactants.

To make the stopped-flow spectrometer anaerobic, the flow-circuit of this apparatus was repeatedly washed with anaerobic buffer. Anaerobic solutions (2 or 4 mL) were prepared in gastight glass syringes (5 or 10 mL, Hamilton, Nevada, USA). NADPH and buffer solutions were made anaerobic by bubbling argon through the solutions for 10 min. To deoxygenate the enzyme solution, argon was blown on the surface of the solution for 10 min. *Aspergillus niger* glucose oxidase (type VII) and glucose were added to the solutions to remove any residual traces of dioxygen (final concentrations 0.5 µM and 2 mM, respectively).

All data were analyzed using the Pro-Data software (Applied Photophysics, Surrey, UK) and plotted with KaleidaGraph software (Synergy Software, Reading, PA). All stopped-flow traces at 355 nm were fit to an exponential function to determine the observed rates (k_{obs}), with a first phase accounting for the formation of the C4a-peroxyflavin intermediate and a second phase accounting for its decay. A double exponential function was also used to fit the stopped-flow traces at 440 nm obtained in the presence of cyclohexanone, with a first phase accounting for 60% of the absorption change and a k_{obs} value similar to the k_{cat} value. In the absence of cyclohexanone, the enzyme was not completely reoxidized after 350 s (Figure 2A), and the corresponding stopped-flow traces at 440 nm were best fit to a single exponential function.

Determination of Melting Temperatures and Long-term Stability. The melting temperature (T_m) value for purified TmCHMO and AcCHMO, fused to PTDH and a His-Tag, was determined using the ThermoFAD method as previously described.[15] For these assays, 10 µM enzyme in the absence or the presence of 0.5 mM $NAD(P)^+$ was prepared in 50 mM Tris-HCl at pH 7.0. These samples were subjected to a temperature gradient. An increase in the fluorescence signal is observed when the FAD becomes solvent-exposed during the denaturation process. The T_m is the maximum of the derivative of the sigmoidal curve obtained by plotting the fluorescence intensity against the temperature.

To compare the long-term stability of TmCHMO and AcCHMO, these enzymes (10 µM) were incubated at 20, 30 and 45 ºC in 50 mM Tris-HCl at pH 7.0 for at least 16 h (final volume 400 µL). Other enzyme solutions were prepared in the same buffer containing 14% acetonitrile and incubated at 20 ºC. An aliquot of each enzyme solution was taken at several time points to determine enzyme activity on cyclohexanone using the NADPH consumption assay described above. Reactions contained 0.1 µM enzyme, 150 µM NADPH, and 30 µM cyclohexanone in air-saturated 50 mM Tris-HCl pH at 7.0 and 25 °C (final volume 1 mL). They were assayed in duplicate.

Conversion of *rac*-Bicyclo[3.2.0]hept-2-en-6-one and Thioanisole using Purified Enzyme. Purified TmCHMO, AcCHMO, and TfPAMO (2 µM) was reacted with either *rac*-bicyclo[3.2.0]hept-2-en-6-one (11 mM) or thioanisole (3.4 mM) at 24 °C and 200 rpm (Innova 44 incubator shaker, New Brunswick). All reactions contained 150 µM NADPH, 20 mM phosphite, and 50 mM Tris-HCl at pH 7.0. Stocks of both substrates were prepared in ethanol, but all reactions only contained 1.2% ethanol. A final reaction volume of 0.5 mL was prepared in a 20 mL vial (headspace, screw top, Agilent). The reaction time for the reactions containing *rac*-bicyclo[3.2.0]hept-2-en-6-one and thioanisole was 20 h and 2 h, respectively. All reactions were analyzed by chiral GC as described below.

After the incubation, the reaction was mixed with 0.5 mL of tert-butyl methyl ether containing 0.1% mesitylene as an internal standard. The resulting solution was vortex-mixed (1 min) and centrifuged (5 min, 20,238 g, room temperature). The top layer was kept, and the bottom layer was extracted again following the same procedure. Anhydrous magnesium sulfate was added to the organic layer to remove the residual water. The supernatant obtained after centrifugation (5 min, 20,238 g, room temperature) was analyzed. Analyses were carried out by chiral GC as described below.

Conversion of 2-Butanone using Purified Enzyme or Whole Cells. Purified TmCHMO and AcCHMO (1 µM) was reacted with 2-butanone (11 mM) at 17 °C and 200 rpm (Innova 44 incubator shaker, New Brunswick). All reactions contained 150 µM NADPH, 20 mM phosphite, and 50 mM Tris-HCl at pH 7.0. A

final reaction volume of 0.5 mL was prepared in a 20 mL vial (headspace, screw top, Agilent). The reaction time was 48 h. Reactions were stopped by incubation at 80 °C for 10 min. The same reactions were run in the presence of various cosolvents (Table S5). All reactions were assayed in duplicate and analyzed by headspace GC-MS as described below.

The conversion of 2-butanone was also investigated using whole cells of NEB 10-*β* *E. coli* expressing either TmCHMO or AcCHMO. *E. coli* cells were grown in 250 µL Luria-Bertani medium containing 50 µg/ml ampicillin (LB-amp) using a 2 mL deep 96-square well plate (Waters) covered by an adhesive seal (AeraSeal film, Excel Scientific). Inoculation of these cultures was carried out using the corresponding glycerol stocks frozen at -80 °C and toothpicks. After 14 h of incubation at 37 °C and 1,050 rpm (Titramax 1000 incubator shaker, Heidolph Instruments), 20 µL from each culture were transferred into the wells of a 2 mL deep 96-square well plate. Subsequently, 180 µL LB-amp containing 0.02% (w/v) L-arabinose and 11 mM 2-butanone were added to each well. The plate was covered with a polypropylene cap mat (Waters) and then incubated for 24 h at 24 °C and 1,050 rpm before carrying out the analyses. All cultures were prepared in triplicate and analyzed by headspace GC-MS as described below.

Headspace GC-MS and Chiral GC Analyses. Enzyme reactions with 2-butanone were analyzed by headspace GC-MS using a GCMS-QP2010 (Shimadzu) in an air-conditioned room (21 °C). A HP-1 column (30 m x 0.25 mm x 0.25 µm, Agilent) was used. 250 µL headspace samples were injected. The injector temperature was set at 150 °C. The temperature program used for the cultures in 96-square well plates and the reactions of purified enzyme in vials was isothermal at 35 °C for 1.7 min and 3.3 min, respectively. Reactions prepared in vials were stirred at 40 °C for 2.5 min before the analysis. Standards were used to identify the substrates and products by retention time and their characteristic fragmentation pattern, which were compared to the results obtained in a previous study.[16] Calibration curves of 2-butanone, ethyl acetate, and methyl propanoate were carried out to quantify these compounds in the reactions.

Enzyme reactions with *rac*-bicyclo[3.2.0]hept-2-en-6-one were analyzed by chiral GC using a 7890A GC System (Agilent Technologies) and a CP Chiralsil Dex CB column (25 m x 0.25 mm x 0.25 µm, Agilent). The temperature program was: i) from 40 ºC to 130 ºC at 10 ºC/min; ii) 130 ºC for 15 min; and iii) from 130 ºC to 40 ºC at 10 ºC/min. Enzyme reactions with thioanisole were analyzed by chiral GC using a 6890 GC System (Hewlett Packard) and a Chiraldex G-TA column (30 m x 0.25 mm x 0.25 µm, Supelco). The temperature program was: i) 35 ºC for 1 min; ii) from 35 ºC to 170 ºC at 10 ºC/min; iii) 170 ºC for 8 min; and iv) from 170 ºC to 35 ºC at 10 ºC/min. In all chiral GC analyses, the inlet temperature was 250 ºC, the volume injected was 3 µl, and the split ratio was 1:50. Substrates and products were identified based on the results obtained in other studies as previously indicated.[17] To confirm the identity of the products obtained in the enzyme reactions with thioanisole, this sulfide was oxidized to the corresponding sulfoxide (methyl phenyl sulfoxide) using hydrogen peroxide and glacial acetic acid at 25 °C as previously described.[18] The retention times for the products obtained in the non-enzymatic reaction of thioanisole were compared to those for the products obtained in the enzyme-catalyzed reactions of this compound.

Protein Crystallization, X-Ray Data Collection, and Structure Determination. Native TmCHMO was crystallized at 293 K using the sitting-drop vapour diffusion technique at 20 °C. Equal volumes of 12 mg mL^{-1} TmCHMO in 20 mM Tris-HCl at pH 7.5 and reservoir solution were mixed. The reservoir solution contained 30% polyethylene glycol (PEG) 3350 in 0.5 M lithium acetate (w/v), 25% PEG 6000 in 0.1 M Tris-HCl at pH 8.5 (w/v), or 30% PEG 4000 in 0.1 M Tris-HCl at pH 8.5 (w/v). Initial conditions were screened using the JSCG, PEG and ammonium sulfate screens (Qiagen) in 96-well sitting drop trays. Crystals were cryoprotected by soaking in the reservoir solution containing 20% glycerol (v/v). When performed, reduction of crystals was achieved by adding 25 µL of the reservoir solution to the drop with crystals. Subsequently, this solution was exchanged to a cryobuffer solution containing 80 mM sodium dithionite. Reduction was evident after the bleaching of the yellow colour of the crystals and was confirmed by microspectrophotometry (Figure S13). X-Ray diffraction data were collected at the PXIII beamline of the Swiss Light Source in Villigen, Switzerland (SLS) and at the BM14-1 beamline of the European Synchrotron Radiation Facility in Grenoble, France (ESRF). The images were integrated and scaled using MOSFLM.[19] Intensities were merged and converted to amplitudes with Aimless[20] and other software of the CCP4 Suite[21] (Table S7). The structures were solved with MOLREP,[22] and the coordinates of

RhCHMO[23] (PDB: 4RG3) as search model. COOT[24] and REFMAC5[25] were employed to carry out alternating cycles of model building and refinement. Figures were created with CCP4mg,[26] VMD[4] and Chimera.[5]

Microspectrophotometry. To investigate if the crystalline TmCHMO remained redox-active, we probed crystals by microspectrophotometry. The crystals exhibited a UV-visible absorbance spectrum very similar to that reported for protein solutions. Soaking the crystals in dithionite solutions led to flavin reduction, which takes place in 1-3 min depending on the crystal size (Figure S13, spectrum a). Dithionite-reduced crystals could be reoxidized in 30-180 s by exposure to aerated solutions (spectra b and c). These absorbance spectra were measured on cryo-cooled crystals in off-line mode at the Cryobench laboratory at beam-line ID29S of ESRF.[27]

5. REFERENCES

[1] F. Sievers, A. Wilm, D. Dineen, T. J. Gibson, K. Karplus, W. Li, R. Lopez, H. McWilliam, M. Remmert, J. Söding, J. D. Thompson, D. G. Higgins, *Mol. Syst. Biol.* **2011**, *7*, 539-544.
[2] N. Saitou, M. Nei, *Mol. Biol. Evol.* **1987**, *4*, 406-425.
[3] S. Kumar, G. Stecher, K. Tamura, *Mol. Biol. Evol.* **2016**, *33*, 1870-1874.
[4] W. Humphrey, A. Dalke, K. Schulten, *Journal of Molecular Graphics* **1996**, *14*, 33-38.
[5] E. F. Pettersen, T. D. Goddard, C. C. Huang, G. S. Couch, D. M. Greenblatt, E. C. Meng, T. E. Ferrin, *Journal of Computational Chemistry* **2004**, *25*, 1605-1612.
[6] a) F. Secundo, S. Fiala, M. W. Fraaije, G. de Gonzalo, M. Meli, F. Zambianchi, G. Ottolina, *Biotechnol. Bioeng.* **2011**, *108*, 491-499; b) G. Carrea, G. Ottolina, S. Riva, *Trends Biotechnol.* **1995**, *13*, 63-70.
[7] P. D. Adams, P. V. Afonine, G. Bunkoczi, V. B. Chen, I. W. Davis, N. Echols, J. J. Headd, L.-W. Hung, G. J. Kapral, R. W. Grosse-Kunstleve, A. J. McCoy, N. W. Moriarty, R. Oeffner, R. J. Read, D. C. Richardson, J. S. Richardson, T. C. Terwilliger, P. H. Zwart, *Acta Crystallographica Section D* **2010**, *66*, 213-221.
[8] F. Korn-Wendisch, F. Rainey, R. M. Kroppenstedt, A. Kempf, A. Majazza, H. J. Kutzner, E. Stackebrandt, *Int. J. Syst. Bacteriol.* **1995**, *45*, 67-77.
[9] D. E. Torres Pazmiño, A. Riebel, J. de Lange, F. Rudroff, M. D. Mihovilovic, M. W. Fraaije, *ChemBioChem* **2009**, *10*, 2595-2598.
[10] M. P. Malakhov, M. R. Mattern, O. A. Malakhova, M. Drinker, S. D. Weeks, T. R. Butt, *J. Struct. Funct. Genomics* **2004**, *5*, 75-86.
[11] H. L. van Beek, H. J. Wijma, L. Fromont, D. B. Janssen, M. W. Fraaije, *FEBS Open Bio* **2014**, *4*, 168-174.
[12] J. Felsenstein, *Evolution* **1985**, *39*, 783-791.
[13] E. Zuckerkandl, L. Pauling, in *Evolving genes and proteins* (Eds.: V. Bryson, H. J. Vogel), Academic Press, New York, **1965**, pp. 97-166.
[14] a) D. Sheng, D. P. Ballou, V. Massey, *Biochemistry* **2001**, *40*, 11156-11167; b) M. W. Fraaije, J. Wu, D. P. Heuts, E. W. Van Hellemond, J. H. L. Spelberg, D. B. Janssen, *Appl. Microbiol. Biotechnol.* **2005**, *66*, 393-400.
[15] F. Forneris, R. Orru, D. Bonivento, L. R. Chiarelli, A. Mattevi, *FEBS J.* **2009**, *276*, 2833-2840.
[16] H. L. van Beek, R. T. Winter, G. R. Eastham, M. W. Fraaije, *Chem Commun* **2014**, *50*, 13034-13036.
[17] H. M. Dudek, G. de Gonzalo, D. E. T. Pazmiño, P. Stępniak, L. S. Wyrwicz, L. Rychlewski, M. W. Fraaije, *Appl. Environ. Microbiol.* **2011**, *77*, 5730-5738.
[18] H. Golchoubian, F. Hosseinpoor, *Molecules* **2007**, *12*, 304-311.
[19] T. G. G. Battye, L. Kontogiannis, O. Johnson, H. R. Powell, A. G. W. Leslie, *Acta Crystallographica Section D* **2011**, *67*, 271-281.
[20] P. R. Evans, G. N. Murshudov, *Acta Crystallographica Section D* **2013**, *69*, 1204-1214.
[21] M. D. Winn, C. C. Ballard, K. D. Cowtan, E. J. Dodson, P. Emsley, P. R. Evans, R. M. Keegan, E. B. Krissinel, A. G. W. Leslie, A. McCoy, S. J. McNicholas, G. N. Murshudov, N. S. Pannu, E. A. Potterton, H. R. Powell, R. J. Read, A. Vagin, K. S. Wilson, *Acta Crystallographica Section D* **2011**, *67*, 235-242.
[22] A. Vagin, A. Teplyakov, *Journal of Applied Crystallography* **1997**, *30*, 1022-1025.
[23] B. J. Yachnin, M. B. McEvoy, R. J. D. MacCuish, K. L. Morley, P. C. K. Lau, A. M. Berghuis, *ACS Chemical Biology* **2014**, *9*, 2843-2851.
[24] P. Emsley, B. Lohkamp, W. G. Scott, K. Cowtan, *Acta Crystallographica Section D* **2010**, *66*, 486-501.
[25] G. N. Murshudov, P. Skubak, A. A. Lebedev, N. S. Pannu, R. A. Steiner, R. A. Nicholls, M. D. Winn, F. Long, A. A. Vagin, *Acta Crystallographica Section D* **2011**, *67*, 355-367.
[26] S. McNicholas, E. Potterton, K. S. Wilson, M. E. M. Noble, *Acta Crystallographica Section D* **2011**, *67*, 386-394.

[27] D. von Stetten, T. Giraud, P. Carpentier, F. Sever, M. Terrien, F. Dobias, D. H. Juers, D. Flot, C. Mueller-Dieckmann, G. A. Leonard, D. de Sanctis, A. Royant, *Acta Crystallographica Section D* **2015**, *71*, 15-26.
